# 

**Published:** 1878-10

**Authors:** 


### Chicago Medical Journal

AND

### EXAMINER.

Vol. XXXVII.—OCTOBER, 1878.—No. 4.

### To our Readers and Correspondents.

Beginning with Vol. XXXVII., July, 1878, the Chicago Medical Journal and Examiner prints all records of length and weight in terms of the Metric System, and all records of temperature in degrees of the Centigrade Scale. The Metric System, legalized in the United States and Great Britain, is extensively or exclusively employed by other civilized nations, and has thus become an essential part of the international language of science. It is recommended for adoption to the profession in this country by the American Medical Association and other scientific bodies. To-day no physician can afford to be ignorant of its value, its simplicity and the meaning of its terms.

The subjoined tables and scales, which have been kindly prepared for us by Prof. W. S. Haines, will be continuously reproduced in subsequent numbers of this journal, for the ready reference of our readers and correspondents.

### Metric Measures of Length.

| | | | |
|---|---|---|---|
| Millimeter | 0.001 | of a Meter | 0.03937 inches. |
| Centimeter | 0.01 | " " | 0.39370 " |
| Decimeter | 0.1 | " " | 3.93707 " |
| **Meter** | **1.** | **Meter** | **39.37079** " |
| Decameter | 10. | Meters | 393.70790 " |
| Hectometer | 100. | " | 3937.07900 " |
| Kilometer | 1000. | " | 39370.79000 " |

### Metrical Weights.

| | | | |
|---|---|---|---|
| Milligram | 0.001 | of a Gram | 0.015 grains. |
| Centigram | 0.01 | " " | 0.154 " |
| Decigram | 0.1 | " " | 1.543 " |
| **Gram** | **1.** | **Gram** | **15.432** " |
| Decagram | 10. | Grams | 154.323 " |
| Hectogram | 100. | " | 1543.234 " |
| Kilogram | 1000. | " | 15434.348 " |

☞ The United States "nickel" five cent piece weighs five grams, and is two centimeters in diameter.

### Approximate Equivalent of Metrical Weights.

*For Rapid Reference.*

| *Milligrams.* | *Grains.* |
|---|---|
| 1 (written 0.001 or \|001)* | $\frac{1}{65}$ |
| 2 | $\frac{1}{32}$ |
| 3 | $\frac{1}{22}$ |
| 4 | $\frac{1}{16}$ |
| 5 | $\frac{1}{13}$ |
| 6 | $\frac{1}{11}$ |
| 7 | $\frac{1}{9}$ |
| 8 | $\frac{1}{8}$ |
| 9 | $\frac{1}{7}$ |

| *Centigrams.* | *Grains.* |
|---|---|
| 1 (written 0.01 or \|01) | $\frac{1}{6}$ |
| 2 | $\frac{1}{3}$ |
| 3 | $\frac{6}{13}$ |
| 4 | $\frac{7}{11}$ |
| 5 | $\frac{3}{4}$ |
| 6 | $\frac{9}{10}$ |
| 7 | 1 |
| 8 | $1\frac{1}{4}$ |
| 9 | $1\frac{1}{3}$ |

A Kilogram=$2\frac{1}{5}$ lbs. Avoirdupois.

| *Decigrams.* | *Grains.* |
|---|---|
| 1 (written 0.1 or \|1) | $1\frac{1}{2}$ |
| 2 | 3 |
| 3 | $4\frac{1}{2}$ |
| 4 | 6 |
| 5 | $7\frac{1}{2}$ |
| 6 | 9 |
| 7 | 11 |
| 8 | $12\frac{1}{2}$ |
| 9 | 14 |

| *Grams.* | *Grains.* |
|---|---|
| 1 (written 1. or 1\|) | 15 |
| 2 | 30 |
| 3 | 46 |
| 4 | 61 |
| 5 | 77 |
| 6 | 92 |
| 7 | 108 |
| 8 | 123 |
| 9 | 139 |

* The decimal *line* instead of points makes errors impossible.

### Metric Fluid Measures.

When using the metric system, fluids are preferably prescribed by weight, employing the gram, its multiples and subdivisions, just the same as with solids, thus avoiding the errors due to refraction, adhesion, and inaccurate measuring vessels. For practical purposes four grams of water may be regarded as equivalent to a fluid drachm of that liquid, and the same may be considered true of tinctures and infusions; syrups, on the average, are about one-third heavier than water, so that a fluid ounce of a syrup will be approximately represented by 43 grams.

If preferred, however, fluids may be prescribed by volume in the metric, just as in the present system, using for that purpose the *Cubic Centimeter*, that is, a volume represented by a cube all of whose sides measure one centimeter. An ordinary back-gammon die is usually about this size. One cubic centimeter (written 1 C. C.) = 16.231 minims. It is approximately regarded as one fourth of a fluid drachm.

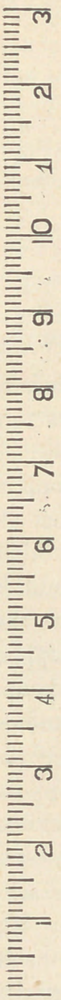

CENTIMETERS AND MILLIMETERS.

#### Approximate Equivalents of Cubic Centimeter.

| | | | | |
|---|---|---|---|---|
| 0.001 | C. C. | = ......... | $^1/_{65}$ | minim. |
| 0.01 | " | = ......... | $^1/_6$ | " |
| 0.1 | " | = ......... | $1^1/_2$ | " |
| **1.** | **C. C.** | **= .................** | **15** | **minim.** |
| 4. | " | = ......... | 1 | fluid drachm. |
| 16. | " | = ......... | 4 | fluid drachms. |
| 32. | " | = ......... | 1 | ounce. |

1000 C. C. (usually known as a **Liter**) is a trifle more than one quart, wine measure.

The following prescription—

R: Potassii bromidi, ℥i.

Elixir aurantii, fl., ℥viij.

M.

Would, in metric terms, be written:

Potassic bromide, 32 |

Orange elixir, 250 |

Mix.

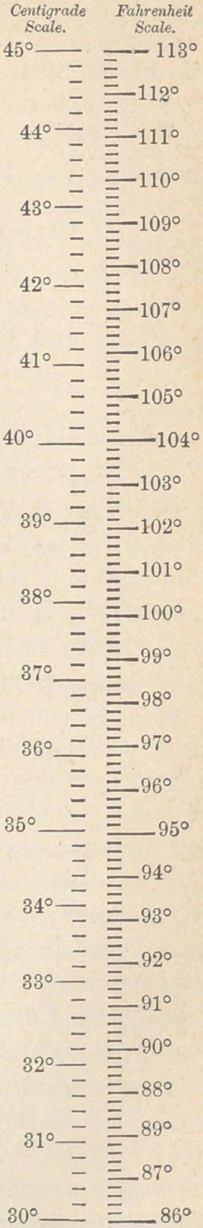

### ANNOUNCEMENTS FOR THE MONTH.

### SOCIETY MEETINGS.

Chicago Medical Society—Mondays, Oct. 7 and 21.
West Chicago Medical Society—Mondays, Oct. 14 and 28.

### CLINICS.

MONDAY.

Eye and Ear Infirmary—2 p. m., Ophthalmological, by Prof. Holmes; 3 p. m., Otological, by Prof. Jones.
Mercy Hospital—1:30 p. m., Surgical, by Prof. Andrews.
Rush Medical College—2 p. m., Dermatological and Venereal, by Dr. Hyde; 3 p. m., Medical, by Dr. Bridge.
Woman's Medical College—2 p. m., Dermatological, by Dr. Maynard.

TUESDAY.

Mercy Hospital—1:30 p. m., Medical, by Prof. Hollister.

WEDNESDAY.

Chicago Medical College—1:30 p. m., Eye and Ear, by Prof. Jones.

THURSDAY.

Eye and Ear Infirmary—2 p. m., Ophthalmological, by Dr. Hotz.
Chicago Medical College—1:30 p. m., Medical, by Prof. Quine.

FRIDAY.

Mercy Hospital—1:30 p. m., Medical, by Prof. Davis.

SATURDAY.

Rush Medical College—2 p. m., Surgical, by Prof. Gunn.
Chicago Medical College—2 p. m., Surgical, by Prof. Isham; 3 p. m., Neurological, by Prof. Jewell.

Daily Clinics, from 2 to 4 p. m., at the Central Free Dispensary, and at the South Side Dispensary.

